# Preferred Women’s Waist-to-Hip Ratio Variation over the Last 2,500 Years

**DOI:** 10.1371/journal.pone.0123284

**Published:** 2015-04-17

**Authors:** Jeanne Bovet, Michel Raymond

**Affiliations:** Institute of Evolutionary Sciences, University of Montpellier, CNRS, IRD, EPHE, CC 065, Place Eugène Bataillon, Montpellier, France; London School of Hygiene and Tropical Medicine, UNITED KINGDOM

## Abstract

The ratio between the body circumference at the waist and the hips (or WHR) is a secondary sexual trait that is unique to humans and is well known to influence men’s mate preferences. Because a woman's WHR also provides information about her age, health and fertility, men's preference concerning this physical feature may possibly be a cognitive adaptation selected in the human lineage. However, it is unclear whether the preferred WHR in western countries reflects a universal ideal, as geographic variation in non-western areas has been found, and discordances about its temporal consistency remain in the literature. We analyzed the WHR of women considered as ideally beautiful who were depicted in western artworks from 500 BCE to the present. These vestiges of the past feminine ideal were then compared to more recent symbols of beauty: *Playboy* models and winners of several *Miss* pageants from 1920 to 2014. We found that the ideal WHR has changed over time in western societies: it was constant during almost a millennium in antiquity (from 500 BCE to 400 CE) and has decreased from the 15^th^ century to the present. Then, based on *Playboy* models and *Miss* pageants winners, this decrease appears to slow down or even reverse during the second half of the 20^th^ century. The universality of an ideal WHR is thus challenged, and historical changes in western societies could have caused these variations in men’s preferences. The potential adaptive explanations for these results are discussed.

## Introduction

Although women’s attractiveness is considered complex and difficult to quantify, various physical traits are now well known to influence men’s mate choice. Among those traits, the waist-to-hip ratio (WHR, the ratio between the body circumference at the waist and the hips), which measures the distribution of body fat, has been the focus of extensive research. Evolutionary theories of human mate selection contend that the WHR is an honest indicator of fertility and is used by men to choose a mate likely to increase their reproductive success. Consistent with this hypothesis, the distribution of body fat provides information about age, health, fertility, and sexual behavior [1–7], and women with an hourglass shape (i.e., a low WHR) are more attractive to men [2, 7–9]. One particular aspect of the literature has focused on the universality of this preference for a low WHR. Several studies show a certain consistency across populations in the preferences of men toward a low WHR [10–14], whereas a preference for a relatively higher WHR has been observed in some other societies [15, 16]. However, this apparent variability could result from the stimuli used, as other studies using different stimuli found a preference for a low WHR [17, 18]. In fact, the stimuli used are not always adapted to the local population, or do not control for the women's body mass [17, 19]. In addition, there is no consensus concerning the consistency of WHR over time. Several studies have used measurements of *Playboy* models and *Miss America* contestants, which are taken as representative icons of western beauty standards. Here again, some studies underline the remarkable consistency of the preferred WHR [20, 21], whereas others insist on the variability of the ideal body shape over the last decades [22–25]. Nevertheless, studies based on data from *Playboy* and *Miss* pageants concern the relatively recent past, with the oldest measurements dating from the 1920s. For the more distant past, indications about physical preferences come from artworks, such as paintings and sculptures. Although many references have been made to ancient artworks, either to underscore the changing ideals of attractiveness in western societies [22, 26], or to prove the invariability of the preferred WHR [3, 6, 27, 28] there is no published quantitative study (to our knowledge) based on a large sample of artworks. This could be due to the difficulty of obtaining measurements of women represented in artworks: it is complex to objectively estimate the WHR of painted women with different postures or orientations. It is easier for sculptures of nude women, although it could be difficult to have direct access to the artworks scattered across museums. In addition, artwork has several purposes, and the depicted women do not always represent the ideal woman body: it is thus important to consider only art items specifically designed to represent attractive women. To our knowledge, this point has never been considered.

This paper is divided into 3 studies. In the first study, we investigated the evolution of WHR from 500 BCE to the present using artworks (painting and sculptures) representing women usually considered as beautiful, such as Aphrodite or Venus. As the human brain has the capacity to judge and very quickly and efficiently compare human bodies [29], even if not normalized for characteristics such as the body posture, a large sample of observers was used to estimate the WHR of women represented in artworks by comparing them to drawings of female silhouettes. In the second study, we analyzed the evolution of WHR from 1920 to 2014 using measurements of *Playboy* models and winners of several *Miss* pageants. In the third study, we collapse these 2 datasets after a calibration step to find the best function describing the evolution of the WHR according to time (from 500 BCE to 2014).

## Study 1

### Materials and Methods

#### Stimuli

216 artworks (160 paintings and 56 sculptures) representing women from 500 BCE to the present were collected from various online open sources (mainly Wikipedia and art gallery websites from various countries). One hundred and fifty of these artworks represented women usually considered as beautiful, such as Aphrodite or Venus (Greek or Roman goddess of love, beauty, pleasure, and procreation), Graces (Greek or Roman goddesses of charm, beauty, nature, human creativity and fertility) and Psyche (a mythological young woman so beautiful that she was compared to Venus), and 66 artworks represented women not specifically famous for their beauty, such as Eve (according to the creation myth of Abrahamic religions, the first woman created by God) and Susanna (a biblical Hebrew woman falsely accused by lecherous voyeurs). In addition to the subject, the only selection criteria was that the selected painting or sculpture should depict the woman at least partially naked, with a sufficient view allowing for the evaluation of her silhouette. For each artwork, the following information was collected: date of creation (divided into periods of 50 years), artist’s name and birthplace, and subject depicted (Aphrodite, Venus, Graces, Psyche, Eve or Susanna). The women’s posture (standing, sitting or lying) and orientation (front, back or profile) were recorded. All of the artists, and their depictions, were Caucasian. If an artist had produced more than one version of the same subject, the artwork allowing the easiest evaluation of the WHR (depending on the view, the woman's posture, the quality of the picture, etc…) was chosen. The resulting sample is certainly not comprehensive, although it is probably representative of what Wikipedia and art galleries possess. Nude women have been represented in art during Classical Antiquity until the end of the fourth century and were then almost completely absent from artwork during millennia due to a ban from the Christian church [30, 31]. Nude women were represented again in artwork during the Renaissance until today. Thus, the collected artworks were divided into an *antique* period (from 500 BCE to 400 CE) and a *recent* period (from 1400 to 2014 CE).

**Fig 1:**
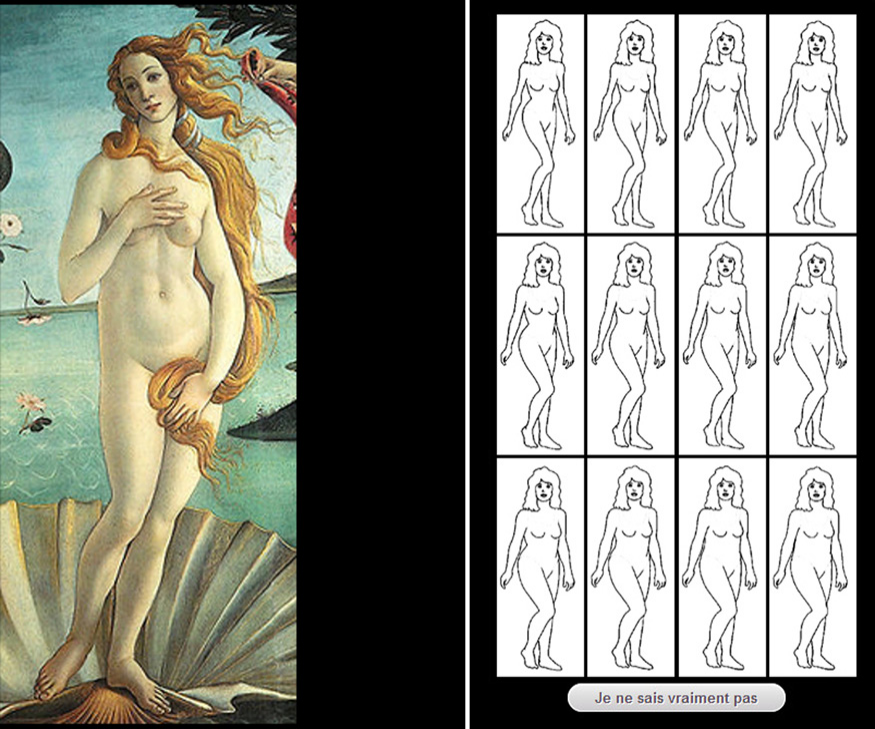
A typical screen shot during the evaluation of the women’s silhouettes in the artworks by the participants in Study 1. An online test was developed to randomly present artworks to raters. On the right part of the screen, 12 drawn figures of women were displayed, representing 3 different weight categories and 4 WHRs within each weight category (from Singh [21]). For each artwork, the rater had to click on the figure that most closely resembled the woman depicted on the artwork according to the rater. A given rater had 17 distinct artworks to assess. Three artworks, which were randomly chosen among those previously observed, were presented again at the end to estimate judgment reliability.

*Procedure*. An online test was developed to randomly present artworks to raters. On the right part of the screen, 12 drawn figures of women representing 3 different body weight categories (underweight, normal weight and overweight) and 4 WHRs within each weight category were displayed (the figures are from Singh [21], see Fig 1). For each artwork, the rater had to click on the figure that most closely resembled the woman depicted on the artwork. A given rater had 17 distinct artworks to assess, which were randomly chosen among the whole dataset. Then, three artworks, which were randomly chosen among the 17 previously observed, were presented again at the end to estimate judgment reliability. A judgment is considered as unreliable if, for the same artwork, the second judgment differs from the first one of more than one figure in each dimension (WHR or weight). Because the task could require some time to be completely understood by the raters, the 4 first judgments of each rater were not included in the analyses. Volunteer raters were unaware of the purpose of the study when assessing artworks. Public advertisements were dispatched in local stores and social networks. These advertisements contained the principle of the test (rating artworks), and a website address. People went to the website on their own will if they wanted to participate. For each rater, only sex, age and nationality were collected. This information could not allow a personal identification (identifying information such as name or IP address were not collected). The content of this experiment (an online evaluation of artworks), didn’t required any specific authorization from an ethics committee. Written consent was not required from the participants for an online survey (their volunteer participation on the web site is equivalent to a tacit consent). Moreover, participants could confirm their willingness to participate to our study through a question displayed at the end of the online test (and only those that agreed where considered for further analyses).

#### Statistical analysis

Because participants had to compare two-dimensional pictures, we directly measured the apparent WHR on the 2D-drawn figures from Singh [21]. These WHR measures were associated with each choice made by raters, and a mean estimated WHR was calculated for each woman. Linear regressions were used to analyze the mean estimated WHR of each woman (numeric variable from 0.70 to 0.92). In the first model, we compared women’s mean WHR for *antique* and *recent* artworks (binary variable). Then, within each period, we analyzed the effect of the date of the artworks’ creation (continuous variable) on women’s WHR. In all models, the type of artwork (painting or sculpture), woman’s posture (standing, sitting or lying) and orientation (front, back or profile) were integrated as confounding variables. A variable “category” (*High attractiveness* if the depicted woman was Venus, Aphrodite, Graces or Psyche, and *Control* if she was Eve or Susanna) was also integrated. As each rater judged only a small fraction of the total number of artwork (13/216 ≈ 6%), each woman was observed by a specific sample of raters. For each artwork, the raters’ mean age and sex ratio were calculated and integrated into the models as confounding variables. To control for potential habituation effects, the mean place of appearance during the test was calculated for each artwork and also integrated into the model as a confounding variable. As each rater judged a random and unique sample of artworks, the expected number of artworks in common to two raters was small (lower than 1), limiting the capacity to compute the inter-rater reliability. Tentatively, Krippendorff’s alpha [32] was used to estimate this value. All statistical analyses were performed using R 2.11.1 (R Core Development Team).

### Results

#### Descriptive statistics

2,164 people responded to the online test. The assessments from non-westerners (according to their declared nationality) and unreliable raters (i.e., raters who did not finish the test or with one or more incorrect answers during the test of judgment reliability) were removed. A total of 1,437 raters were retained in the final sample (mean age 37.4, age range: 18–90, 923 females). Each artwork was evaluated by an average of 75.02 raters (range: 50–118). The inter-rater reliability, estimated by Krippendorff’s alpha, was equal to 0.26.

#### Artworks WHR

The women’s mean estimated WHR varied from 0.715 to 0.883. There was no significant difference between the WHRs of *antique* and *recent* artworks (mean WHR = 0.798 for both, *P* = 0.19). Within the *antique* artworks, there was no significant effect of the date of creation on the WHR (*P* = 0.40, see Table 1). There was no effect of the artwork type (painting or sculpture), woman’s posture (standing, sitting or lying) or orientation (face, back or profile) (*P* >0.4 for all variables). Within the *recent* artworks, there was a significant linear and quadratic effect of the date of creation on the WHR (linear effect: *β* = 0.13, *P* = 0.0020; quadratic effect: *β* = -0.0041, *P* = 0.0011): the artworks’ WHR decreased over time during the *recent* period (1400–2000). There was a significant difference between *the high attractiveness* and the *control* women: *high attractiveness* women had a lower WHR than the *control* women (*β* = -0.014, *P* = 0.010). There was a significant effect of the woman’s posture: sitting women were rated as having a higher WHR than standing or lying women (*β* = 0.012, *P* = 0.031). There was no effect of the artwork type (*P* = 0.060) or woman’s orientation (*P* >0.1). In all the models, the effects of the sample of raters’ characteristics (sex ratio and mean age) and the artwork mean place of appearance during the test were not significant (*P* >0.2).

**Table 1:** Results for study 1.

Variable (value)		Estimate	*P*-value
**Antique artworks**			
Intercept		0.88	<0.001
Date of creation		-0.0017	0.40
Artwork type (painting)		0.020	0.44
Woman's posture	(sitting)	0.011	0.56
	(lying)	-0.018	0.41
Woman's orientation	(back)	-0.019	0.47
	(profile)	-0.00016	0.99
Raters' sex ratio		-0.077	0.47
Raters' mean age		0.0013	0.64
Mean place of appearance		-0.0082	0.46
**Recent artworks**			
Intercept		-0.46	0.25
Date of creation		0.13	0.0020
(Date of creation)2		-0.0041	0.0011
Artwork type (painting)		0.012	0.060
Woman's posture	(sitting)	0.012	0.031
	(lying)	-0.0074	0.26
Woman's orientation	(back)	-0.013	0.14
	(profile)	0.0083	0.10
Category (high attractiveness)		-0.014	0.010
Raters' sex ratio		0.048	0.25
Raters' mean age		0.00070	0.52
Mean place of appearance		0.0041	0.42

Linear regressions were used to analyze the mean estimated WHR of each woman (numeric variable from 0.70 to 0.92). Within each period, we analyzed the effect of the date of the artworks’ creation on women’s WHR. In all models, the type of artwork (painting or sculpture), woman’s posture (standing, sitting or lying) and orientation (front, back or profile) were integrated as confounding variables. A variable “category” (*High attractiveness* if the depicted woman was Venus, Aphrodite, Graces or Psyche, and *Control* if she was Eve or Susanna) was also integrated. For each artwork, the raters’ mean age and sex ratio were calculated and integrated into the models as confounding variables. To control for potential habituation effects, the mean place of appearance during the test was calculated for each artwork and also integrated into the model as a confounding variable.

## Study 2

### Materials and Methods

Data on the WHR of *Playboy* centerfold models from 1953 to 2001 are available in Freese [24] and the *Playboy* corporation website. We collected the measurements of centerfold models from 2001 to July 2014 from the *Playboy* corporation website and Wikipedia. Data for the *Miss America* winners from 1921 to 1986 (when the pageant stopped collecting this information) are available in [24]. When available online, we also collected measurements of the *Miss Universe*, *Miss World* and *Miss Earth* pageant winners. Linear regressions were used to study WHR according to time.

### Results

**Fig 2:**
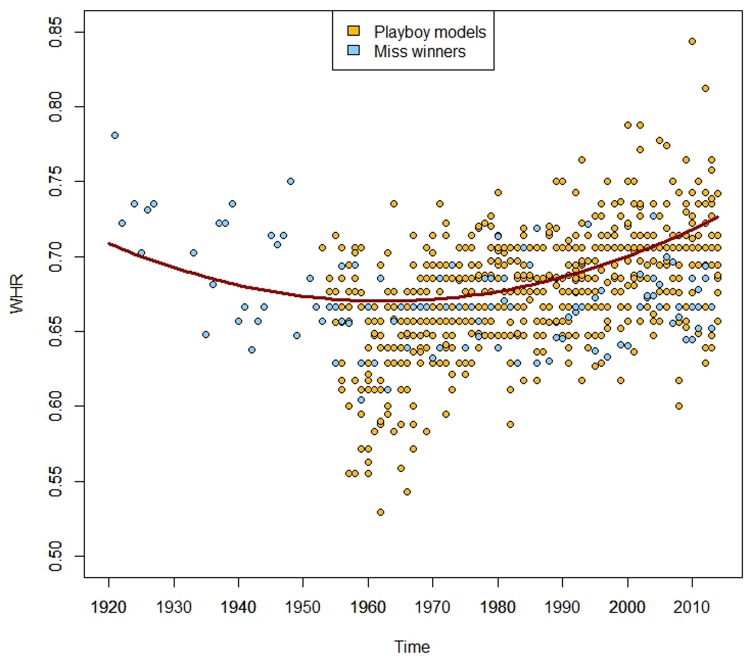
WHR values for *Playboy* centerfold models (in yellow) and winners of 4 *Miss* pageants (*Miss America*, *Miss World*, *Miss Earth and Miss Universe*, in blue) by time of magazine appearance or victory. The data were fit by a linear regression that includes a quadratic term (in red).

During the period from 1921–2014, the *Playboy* models and *Miss* pageants winners’ WHR ranged from 0.529 (for Mickey Winters, appeared in *Playboy* centerfold of Sept. 1962, 45.7 cm/86.3 cm) to 0.844 (for Ashley Hobbs, appeared in *Playboy* centerfold of Dec. 2010, 68.5 cm/81.2 cm), with a mean of 0.677. The data were better fit by a model when including a quadratic term: the women’s WHR had a curvilinear relationship with time (*β* = -0.083 and *β* = 2.12x10^-5^ for time and time^2^, respectively, *P* <0.0001, see Fig 2).

## Study 3

### Materials and Methods

We created a new dataset including the WHRs of artworks, *Playboy* centerfold models and the winners of *Miss* pageants.

#### Calibration of artworks WHR

In study 1, we demonstrated the effect of the artwork type and woman’s posture and orientation on the participants’ estimation of the WHR. The estimated WHRs were thus normalized to make them correspond to a front view standing sculpture. To estimate the validity of the participants’ visual estimation of WHR, we directly measured the WHR of 13 sculptures of standing women (11 *high attractiveness* and 2 *controls*) located in public places and museums (the *Musée des Moulages* in Montpellier and the *Musée Archéologique* in Marseille). Then, for each one of the 13 sculptures, we compared the “real WHR” to the mean WHR estimated by the participants. The mean ratio between the real and estimated WHRs was used to calibrate all the WHRs estimated by the participants.

#### Parameterization and likelihood

According to study 1 and 2, the WHR of artworks was constant during the *antique* period (from 500 BCE to 400 CE) and decreased during the *recent* period (from 1400 to 2014). Study 2 showed that the *Playboy* models and *Miss* winners’ WHR had a curvilinear relationship with time. Thus, the evolution of the WHR according to time *T* on the whole dataset was modeled as follows:

- during the *antique* period: WHR = *M*_0_

where *M*_0_ is the mean corrected WHR during the *antique* period

- during the *recent* period: WHR = *f*(T)

where *f* (T) described the change of WHR according to time in the *recent* period, which include artworks after 1400, the *Playboy* models and winners of *Miss* pageants.

Various forms have been explored for this function, notably:

- *f*(T) = *α* + *β*T
- *f*(T) = *α* + *β*T + *γ*T^2^
- *f*(T) = *α* + *β*T + *γ*T^2^ + δT^3^
- *f*(T) = *α* - *e* ^*βT-γ*^

The likelihood of sampling the WHR observed in our dataset at date *i* (WHR_i_) is as follows:

L_1_ = -Σ_*i*_(WHR_*i*_ - *M*_0_)^2^ for the *antique* period
L_2_ = -Σ_*i*_(WHR_*i*_ - *f*(i))^2^ for the *recent* period

And the global likelihood over the two periods is as follows:

L = L_1_ + L_2_

Maximization of the likelihood function was performed using the optimization method of Byrd et al. [33], which uses a limited-memory modification of the quasi Newton algorithm. For each parameter set, 100 runs were performed, each starting with a random set of initial values to increase the chances of finding the true maximum. All statistical analyses were performed using R 2.11.1 (R Core Development Team).

### Results

#### Calibration of artworks WHR

The mean ratio between the 13 WHRs directly measured and the WHRs estimated for the same sculptures was 1.079 (range 1.018–1.221): on average, participants overestimated sculptures’ WHR by 7.9%. Once corrected for the artwork type, women’s posture and orientation, and participants’ overestimation, the artworks’ WHR varied from 0.655 (*The Awakening of Psyche* by Seignac, 1904) to 0.801 (*Susanna and the Elders* by Pontius, 17^th^ century), with a mean of 0.733.

#### Model selection

**Fig 3:**
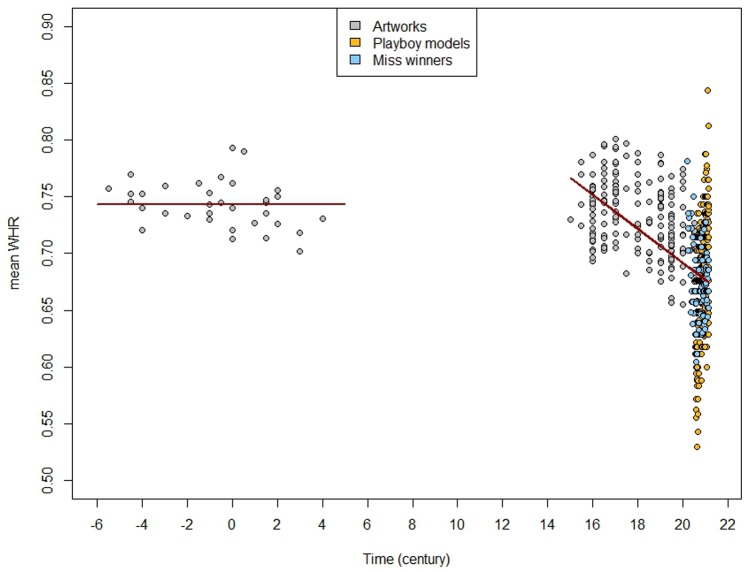
Plot of WHR values for artworks (in gray), *Playboy* centerfold models (in yellow) and winners of *Miss* pageants (in blue) according to time. The best model parameterization was as follows: WHR = 0.74 during the *antique* period and WHR = 0.99 *-* 0.015*T during the *recent* period (in red).

For the full dataset including artworks, *Miss* pageants winners and *Playboy* models, the best model was A, with an AIC of 8.89 (see Fig 3). Models B, D (displaying the same AIC of 10.97), and C (AIC = 12.91) were rejected. The retained parameterization was therefore:

WHR = 0.74 during the *antique* period,

and WHR = 0.99–0.015×T during the *recent* period,

with T in century units, varying from 15 to 20.14. Thus, the WHR of women considered as beautiful was constant during the *antique* period, and then, after a period with no nude women depicted, the preferred WHR decreased from the 15-16^th^ centuries until the present.

## Discussion

This is the first study to analyze men’s preferences on a broad time scale, with a large number of artworks. By choosing artworks representing Greek or Roman goddesses (study 1), we restricted the data to western societies. This choice permitted controlling for the artists’ willingness to represent the most beautiful women, which is often harder to prove for ancient artworks from other cultures. Likewise, the so-called “palaeolithic Venus*”* figurines were not included in our study because the intended representation remains unknown (see [34, 35] for the variety of possible interpretations, spanning from goddess to dolls to symbols of fertility and female power). The fact that, in our study, the represented goddess of beauty (*High attractiveness* type) had a lower WHR than other women, such as Eve or Suzanna (*control* type), underlines the importance of knowing the meaning of the artwork before their interpretation.

We showed that men’s preference for women’s WHR was remarkably constant from 500 BCE to 400 CE, with a mean WHR of 0.74. Then, the WHR considered as ideal decreased from ca 1400 to the present, with a mean WHR of 0.68 for the beginning of the 21st century. Due to the lack of data between 400 and 1400 (due to a ban from the Christian church to depict nude women in art [30, 31], it is difficult to infer exactly when this decrease began and if other variations occurred during this millennium gap.

Even if during the whole period considered here the preferred WHR remained close to the value of 0.7 usually indicated as the ideal WHR in literature (mean for artworks in our study: 0.73) and remained within the normal and fertile range of feminine WHR (range for artworks in our study: 0.65–0.80, normal range of western women: 0.67–0.80 [36]), there was a significant change of men’s preferences during the last 500 years. This result shows that, contrary to the assertion that the preference for WHR is universal and temporally invariant [3, 6, 21, 36], the preferred WHR has changed over time. Moreover, in contrast with the common claim that the ideal shape for women has dramatically changed in the last 20–50 years with the occurrence of mass media, the shift of the beauty standards (at least for the WHR) seems to be older, as the preferred WHR began to decrease since the 15^th^ century.

According to evolutionary theory, men should prefer a female body shape that signals a mate’s high potential to contribute to his reproductive success. However, the usefulness of attention to different signals of mate quality may vary depending on the environment, selecting for facultative preferences that respond to environmental variation [37]. In this way, men’s preference for women’s BMI vary considerably among populations [16, 38–41], with low-resource men preferring a significantly heavier partner than high-resource men [42]. Some studies suggest that a similar link between environment and men’s preferences might exist for the WHR, with men that live in harsher environments preferring a higher WHR [15, 16]. However, this could be an artifact related to the applied methodology, as other studies found a preference for a low WHR in these societies [17, 18]. Moreover, men’s facial femininity preferences correlate positively with the health of the nation [43]. As femininity is associated with lower ratings of dominance [44], this suggests that in harsher environments men prefer cues to resource acquisition and holding potential over high fecundity [43]. The men’s changing preferences concerning the WHR in our study could thus be explained by a change of environment in western societies during the period 1400–2000 [25]. In fact, during this period, the life expectancy, food availability and gross domestic product (GDP) increased in western populations [45–47]. Thus, with an environment becoming relatively more clement, with higher resources and better health, men’s preferences could have evolved toward a lower—thus a more feminine—WHR, favoring cues of fecundity over resource acquisition and holding potential. However, the majority of artworks were produced by relatively wealthy artists for extremely wealthy aristocrats, which reduce the potential role of resources availability on preferences variation.

Similarly, the remarkable consistency of the WHR represented in artworks during the antique period (500 BCE—400 CE) could be linked to the relative consistency of the environment concerning health and resources in the society. However, the similarity of the women represented during this period may be amplified by the fact that artwork imitation was more widespread then, e.g., many Roman statues are Greek copies. This was controlled in this study as much as possible, as statues known to be a copy of an older model were not considered. Nevertheless, it cannot be excluded that widespread artwork imitation at this time, or the restricted number of artworks that remained from this period (e.g., none of the paintings from the Antique Greece have survived), contributes to the apparent stability of the ideal WHR.

The WHR of only *Playboy* models and *Miss* pageant winners (study 2) first decreased and then increased since the 1960s. It is possible that some change in western society, which remains to be identified, has induced this new trend. Interestingly, social inequalities have followed a similar trend during the 20th century, with an initial decrease reflecting an evolution toward more equalities, and then from the seventies, with an increase toward more wealth inequalities [48]. Nevertheless, this phenomenon takes place on a small time frame (~50 years), and it is difficult to compare to the general trend observed over half a millennium. Moreover, this type of fluctuation may have occurred before but was not detected here due to the scale of our sampling.

Alternatively, it has been proposed that the variation of the preferred WHR could result from a another phenomenon: males may possess psychological adaptations that generate a preference depending of the perceived local distribution of WHR in the population, this perceived distribution being a function of the normal female range to which a man is exposed to [49]. A change of the preferred WHR is thus expected when the perceived mean or range of WHR in the population varies. The recent increase in women’s average WHR in western populations [50, 51] could explain the increase in the ideal WHR since the 1960s. Indirect evidence suggest that the mean WHR has changed since the 15^th^ century, for example during the demographic transition (the number of pregnancies affects WHR), and more generally when there was a change in a factor affecting the WHR, such as diet or parasite loads. Population data on WHRs before the 1960s are required to further evaluate this hypothesis.

It is also possible that the women depicted in the present sample of artworks are not a perfect indicator of men's preferences at a given time. The choice of the artworks (representations of goddess of beauty) allows us to be relatively confident concerning the artists' objective. But the ideal of beauty of the artists (or their buyers) may not be representative of the preferences of the rest of the population. Similarly, while the physical appearance of the *Miss* pageant winners and the models of *Playboys* is appreciated by the pageants audience and the magazine readership, it nonetheless remains necessary to establish their validity as ideals for a broader public.

The current medical recommendation for good health is a WHR below 0.85 for women [52]. After calibration, no woman in our study showed a WHR higher than 0.85 (maximum of 0.844, see Study 2). Do the variations within the healthy range of WHR observed here still correspond to fitness differences? The relationship between WHR and fertility is probably continuous, suggesting that a variation (even slight) of WHR translates in a corresponding variation of expected fertility. In fact, a 0.1 unit increase in WHR led to a 30% decrease in probability of conception through artificial insemination [53]. Furthermore, even small variations of WHR (<0.05) are detected by men and influence women's attractiveness [54, 55]. It suggests that the variation of the ideal WHR observed in our study—even within a relatively narrow range (0.529–0.844)—could be adaptive.

In this study, we demonstrated that the WHR of women considered as symbols of beauty did not vary during the antiquity period (500 BCE—400 CE) and decreased since (at least) the 15^th^ century in western societies. A closer analysis of *Playboy* models and *Miss* pageant winners' measurements from 1920 to 2014 revealed a reduction, or even a reversion, of this WHR decrease. The universality of one preferred WHR is thus challenged, and the evolution of men's preferences could be linked to demographic, economic, health or social changes in western societies, which are older than the mass media growth of the 20^th^ century.

Our work shows the necessity to conduct more historical comparisons, which might provide a novel and interesting extension of existing cross-cultural studies about attractiveness [56, 57]. The cognitions and motivation of our ancestors are enshrined in art, which thus comprises a rich data source for cross-generational studies.

## Supporting Information

S1 Table Artworks used in this study. This table presents, for each artwork, its title, author and date of creation, the identity of the woman depicted, her posture and her orientation. The uncorrected WHRs correspond to the women’s mean WHRs estimated by the participants by comparing them to the drawn figures. The corrected WHRs correspond to the estimated WHRs normalized for the type of artwork, the woman’s posture and orientation, and for the raters’ estimation bias.
(PDF)
