# Supplementary material for: Preferred Women’s Waist-to-Hip Ratio Variation over the Last 2,500 Years
Source: PLoS One. 2015 Apr 17;10(4):e0123284. doi: 10.1371/journal.pone.0123284 (PMC4401783; doi:10.1371/journal.pone.0123284)
Supplement: S1 Table — This table presents, for each artwork, its title, author and date of creation, the identity of the woman depicted, her posture and her orientation. The uncorrected WHRs correspond to the women’s mean WHRs estimated by the participants by comparing them to the drawn figures. The corrected WHRs correspond to the estimated WHRs normalized for the type of artwork, the woman’s posture and orientation, and for the raters’ estimation bias. (PDF) [file pone.0123284.s001.pdf]

Artworks	Artist	Century	Subject	Type	Posture	Orientation	Uncorrected WHR	Corrected WHR
The three Graces	Anonymous	-5.5	Grace	sculpture	standing	front	0.810	**0.757**
Aphrodite of Syracus	Praxitele	-4.5	Venus	sculpture	standing	front	0.806	**0.753**
Aphrodite of Cnidus	Praxitele	-4.5	Venus	sculpture	standing	front	0.798	**0.746**
Venus of the Capitol	Praxitele	-4.5	Venus	sculpture	standing	front	0.823	**0.769**
Aphrodite of Braschi	Praxitele	-4	Aphrodite	sculpture	standing	front	0.771	**0.721**
Aphrodite of Cnidus	Praxitele	-4	Aphrodite	sculpture	standing	mean	0.791	**0.740**
Venus of Arles	Praxitele	-4	Aphrodite	sculpture	standing	front	0.805	**0.753**
Crouching Venus	Anonymous	-3	Aphrodite	sculpture	sitting	profile	0.804	**0.736**
Aphrodite with a dolphin	Anonymous	-3	Aphrodite	sculpture	standing	mean	0.813	**0.760**
Aphrodite of Amisos	Anonymous	-2	Aphrodite	sculpture	lying	front	0.784	**0.733**
Venus de Milo	Anonymous	-1.5	Venus	sculpture	standing	mean	0.815	**0.762**
Medici Venus	Anonymous	-1	Aphrodite	sculpture	standing	mean	0.787	**0.736**
Medici Venus	Anonymous	-1	Aphrodite	sculpture	standing	front	0.806	**0.754**
Aphrodite	Antistios	-1	Aphrodite	sculpture	standing	front	0.781	**0.730**
Aphrodite	Diphilos	-1	Aphrodite	sculpture	standing	front	0.795	**0.743**
Aphrodite of Soli	Anonymous	-0.5	Venus	sculpture	standing	front	0.821	**0.767**
Aphrodite of Menophantos	Menophantos	-0.5	Venus	sculpture	standing	front	0.797	**0.745**
Aphrodite	Anonymous	0	Venus	sculpture	standing	mean	0.849	**0.793**
Aphrodite	Anonymous	0	Venus	sculpture	standing	mean	0.816	**0.762**
The Toilet of Venus	Anonymous	0	Aphrodite	sculpture	standing	mean	0.792	**0.740**
Aphrodite of Artemonos	Artemonos	0	Aphrodite	sculpture	sitting	front	0.788	**0.721**
Aphrodite of Cnidus	Diphilos	0	Aphrodite	sculpture	standing	front	0.763	**0.713**
Aphrodite and Eros	Molas	0.5	Aphrodite	sculpture	standing	front	0.845	**0.790**
Venus anadyomene	Anonymous	1	Venus	painting	lying	front	0.791	**0.727**
Crouching Venus	Anonymous	1.5	Aphrodite	sculpture	sitting	front	0.814	**0.745**
Aphrodite	Anonymous	1.5	Venus	sculpture	standing	mean	0.763	**0.714**
Venus of Arles	Anonymous	1.5	Venus	sculpture	standing	back	0.767	**0.735**
Venus of Amiens	Velazquez	1.5	Venus	sculpture	standing	front	0.799	**0.747**
Crouching Venus	Anonymous	2	Aphrodite	sculpture	sitting	profile	0.794	**0.726**
Crouching Venus	Anonymous	2	Aphrodite	sculpture	sitting	profile	0.825	**0.755**
Venus of Esquilin	Anonymous	2	Venus	sculpture	standing	mean	0.803	**0.751**
Aphrodite with a turtle	Anonymous	3	Venus	sculpture	standing	front	0.751	**0.702**

The Birth of Venus	Anonymous	3	Venus	painting	lying	front	0.782	**0.718**
Venus	Anonymous	4	Venus	painting	sitting	profile	0.813	**0.731**
Adam and Eve	Masolino	15	Eve	painting	standing	front	0.795	**0.730**
The Birth of Venus	Botticelli	15.5	Venus	painting	standing	front	0.789	**0.724**
Primavera	Botticelli	15.5	Grace	painting	standing	back	0.830	**0.781**
Adam and Eve	Cleve	15.5	Eve	painting	standing	front	0.809	**0.743**
Venus	Credi	15.5	Venus	painting	standing	front	0.837	**0.770**
Venus and Cupid with a Satyr	Allegri	16	Venus	painting	lying	profile	0.813	**0.747**
Aphrodite of Modesty	Anonymous	16	Venus	sculpture	standing	front	0.773	**0.722**
The three Graces	Baldung	16	Grace	painting	standing	front	0.765	**0.702**
Venus and Cupid	Baldung	16	Venus	painting	standing	front	0.755	**0.693**
Adam and Eve	Baldung	16	Eve	painting	standing	front	0.786	**0.722**
Venus, Cupid, Folly and Time	Bronzino	16	Venus	painting	sitting	front	0.799	**0.718**
Adam and Eve	Coxie	16	Eve	painting	standing	front	0.823	**0.757**
The three Graces	Cranach	16	Grace	painting	standing	front	0.774	**0.711**
Venus callipyge	Cranach	16	Venus	painting	standing	profile	0.793	**0.728**
Adam and Eve	Cranash	16	Eve	painting	standing	profile	0.793	**0.729**
Venus	Cranash	16	Venus	painting	standing	profile	0.776	**0.713**
Venus and Cupid	da Pontormo	16	Venus	painting	lying	front	0.856	**0.787**
Judgment of Paris	di Benvenuto	16	Venus	painting	standing	front	0.811	**0.745**
Adam and Eve	Durer	16	Eve	painting	standing	front	0.816	**0.750**
Adam and Eve	Durer	16	Eve	painting	standing	front	0.810	**0.744**
Venus frustrated	Fiorentino	16	Venus	painting	standing	front	0.810	**0.744**
Adam and Eve	Gossaert	16	Eve	painting	standing	front	0.813	**0.747**
Venus of Urbin	le Titien	16	Venus	painting	lying	profile	0.826	**0.760**
Venus anadyomene	le Titien	16	Venus	painting	standing	profile	0.856	**0.787**
Adam and Eve	Leyden	16	Eve	painting	standing	front	0.838	**0.770**
Susanna and the Elders	Mazzucchelli	16	Susanna	painting	standing	profile	0.825	**0.759**
Adam and Eve	Michelangelo	16	Eve	painting	sitting	profile	0.825	**0.742**
Venus and Cupid at Vulcan's Forge	Penni	16	Venus	painting	standing	front	0.780	**0.716**
Adam and Eve	Raphael	16	Eve	painting	standing	front	0.799	**0.734**
The three Graces	Raphael	16	Grace	painting	standing	front	0.822	**0.755**
Adam and Eve	Robbia	16	Eve	sculpture	standing	front	0.791	**0.739**

Cupid and Psyche	Romano	16	Psyche	painting	sitting	profile	0.792	**0.711**
Adam and Eve	Scorel	16	Eve	painting	standing	front	0.759	**0.696**
Venus and Cupid	Scorel	16	Venus	painting	sitting	front	0.784	**0.704**
Susanna and the Elders	Allori	16.5	Susanna	painting	sitting	profile	0.827	**0.745**
Venus and Cupid	Allori	16.5	Venus	painting	lying	front	0.791	**0.726**
Judgment of Paris	Bertoja	16.5	Venus	painting	standing	profile	0.818	**0.752**
Adam and Eve	Broeuck	16.5	Eve	sculpture	standing	profile	0.766	**0.716**
Venus and Adonis	Cambiaso	16.5	Venus	painting	standing	front	0.838	**0.771**
Venus and Cupid	De Backer	16.5	Venus	painting	standing	front	0.818	**0.752**
Psyche	De Vries	16.5	Psyche	sculpture	standing	front	0.752	**0.703**
Susanna and the Elders	Giuseppe	16.5	Susanna	painting	standing	front	0.812	**0.746**
Venus and Cupid	Limosin	16.5	Venus	painting	lying	front	0.769	**0.706**
Susanna and the Elders	Metsys	16.5	Susanna	painting	sitting	front	0.797	**0.716**
Susanna and the Elders	Palma	16.5	Susanna	painting	sitting	profile	0.818	**0.736**
Susanna and the Elders	Reymond	16.5	Susanna	painting	sitting	profile	0.860	**0.775**
Adam and Eve	Rubens	16.5	Eve	painting	standing	front	0.768	**0.705**
Venus and Cupid	Sustris	16.5	Venus	painting	lying	profile	0.825	**0.758**
Susanna and the Elders	Tintoret	16.5	Susanna	painting	sitting	profile	0.881	**0.795**
Adam and Eve	Tiziano	16.5	Eve	painting	standing	front	0.813	**0.747**
Black Venus	van der Schardt	16.5	Venus	sculpture	standing	front	0.835	**0.780**
Mars et Venus reunis par cupidon	Veronese	16.5	Venus	painting	standing	front	0.866	**0.797**
The three Graces	Von Aachen	16.5	Grace	painting	standing	front	0.777	**0.713**
Judgment of Paris	von Aachen	16.5	Venus	painting	standing	front	0.831	**0.764**
Susanna and the Elders	Blanchard	17	Susanna	painting	sitting	front	0.854	**0.770**
Vénus et les Grâces surprises par un mortel	Blanchard	17	Venus	painting	lying	front	0.810	**0.744**
Susanna and the Elders	Burg	17	Susanna	painting	sitting	front	0.814	**0.732**
Susanna and the Elders	Chauveau	17	Susanna	painting	sitting	front	0.879	**0.793**
Sleeping Venus	Gentileschi	17	Venus	painting	lying	profile	0.781	**0.717**
Susanna and the Elders	Gentileschi	17	Susanna	painting	sitting	front	0.785	**0.705**
Venus after her bath	Giambologna	17	Venus	sculpture	standing	profile	0.782	**0.730**
Susanna and the Elders	Goltzius	17	Susanna	painting	lying	front	0.847	**0.779**
Venus and Adonis	Goltzius	17	Venus	painting	sitting	front	0.790	**0.710**
Susanna and the Elders	Honthorst	17	Susanna	painting	standing	profile	0.863	**0.793**

Adam and Eve	Paggi	17	Eve	painting	sitting	profile	0.829	**0.746**
Susanna and the Elders	Pontius	17	Susanna	painting	standing	profile	0.870	**0.801**
Venus and Mars	Poussin	17	Venus	painting	lying	profile	0.796	**0.731**
Susanna and the Elders	Rembrandt	17	Susanna	painting	sitting	profile	0.819	**0.737**
Reclining Venus with Cupid	Reni	17	Psyche	painting	lying	front	0.792	**0.728**
Susanna and the Elders	Reni	17	Susanna	painting	sitting	profile	0.840	**0.756**
Judgment of Paris	Rubens	17	Venus	painting	standing	front	0.830	**0.763**
Susanna and the Elders	Rubens	17	Susanna	painting	sitting	front	0.866	**0.781**
The three Graces	Rubens	17	Grace	painting	standing	back	0.820	**0.772**
Susanna and the Elders	Stanzione	17	Susanna	painting	sitting	front	0.838	**0.754**
Susanna and the Elders	Stella	17	Susanna	painting	sitting	profile	0.843	**0.759**
Cupid and Psyche	van Dyck	17	Psyche	painting	lying	front	0.827	**0.760**
Venus asks Vulcan to cast arms for her son Aeneas	van Dyck	17	Venus	painting	standing	front	0.871	**0.801**
Venus, Adonis and Cupid	van Ravesteyn	17	Venus	painting	sitting	profile	0.848	**0.764**
Adam and Eve	Zampieri	17	Eve	painting	sitting	front	0.836	**0.753**
Crouching Venus	Coysevox	17.5	Venus	sculpture	sitting	profile	0.827	**0.757**
Mars and Venus in the Forge of Vulcan	Giordano	17.5	Venus	painting	lying	front	0.780	**0.717**
Venus punishing Psyche	Giordano	17.5	Psyche	painting	standing	front	0.855	**0.786**
Susanna and the Elders	Jordaens	17.5	Susanna	painting	sitting	front	0.883	**0.797**
The Toilet of Venus	Vargas	17.5	Venus	painting	lying	back	0.724	**0.682**
A Triumph of Venus	Boucher	18	Venus	painting	sitting	profile	0.826	**0.744**
Abandoned Psyche	Coypel	18	Psyche	painting	sitting	profile	0.785	**0.705**
Susanna and the Elders	Coypel	18	Susanna	painting	sitting	front	0.806	**0.724**
Susanna and the Elders	La Fosse	18	Susanna	painting	sitting	profile	0.854	**0.769**
Venus and Nymphs Bath	Lagrenee	18	Venus	painting	standing	back	0.757	**0.713**
Venus	Pigalle	18	Venus	sculpture	sitting	front	0.831	**0.761**
Susanna and the Elders	Pittoni	18	Susanna	painting	sitting	profile	0.839	**0.755**
Venus and Mars	Pittoni	18	Venus	painting	sitting	profile	0.805	**0.723**
Susanna and the Elders	Ricci	18	Susanna	painting	standing	front	0.849	**0.780**
Adam and Eve	Santerre	18	Eve	painting	standing	front	0.763	**0.700**
Susanna and the Elders	Santerre	18	Susanna	painting	sitting	profile	0.808	**0.726**
Judgment of Paris	Troy	18	Venus	painting	standing	front	0.857	**0.788**
Judgment of Paris	Watteau	18	Venus	painting	standing	back	0.761	**0.717**

The three Graces	Antoine	18.5	Grace	sculpture	standing	front	0.779	**0.728**
Psyche Revived by Cupid's Kiss	Canova	18.5	Psyche	sculpture	lying	front	0.749	**0.700**
Venus	Coustou	18.5	Venus	sculpture	standing	front	0.816	**0.763**
Abandoned Psyche	David	18.5	Psyche	painting	sitting	profile	0.808	**0.726**
Cupid and Psyche	Gerard	18.5	Psyche	painting	sitting	profile	0.772	**0.693**
Adam and Eve	Marie	18.5	Eve	painting	sitting	profile	0.828	**0.746**
Cupid and Psyche	Michel	18.5	Psyche	painting	standing	front	0.764	**0.701**
Abandoned Psyche	Pajou	18.5	Psyche	sculpture	sitting	front	0.750	**0.685**
Susanna and the Elders	Vien	18.5	Susanna	painting	sitting	front	0.812	**0.730**
Susanna and the Elders	Beauvallet	19	Susanna	sculpture	standing	front	0.793	**0.741**
Adam and Eve	Blake	19	Eve	painting	standing	profile	0.833	**0.766**
The three Graces	Canova	19	Grace	sculpture	standing	back	0.784	**0.751**
Venus victrix	Canova	19	Venus	sculpture	lying	front	0.766	**0.716**
Susanna and the Elders	Chasseriau	19	Susanna	painting	standing	profile	0.855	**0.787**
Venus anadyomene	Chasseriau	19	Venus	painting	standing	profile	0.744	**0.683**
Venus in a shell	Christen	19	Venus	sculpture	sitting	profile	0.810	**0.741**
Adam and Eve	Dubufe	19	Eve	painting	sitting	front	0.771	**0.692**
Susanna and the Elders	Galland	19	Susanna	painting	sitting	profile	0.864	**0.779**
Ballerina Carlotta Chabert as Venus	Hayez	19	Venus	painting	standing	back	0.757	**0.712**
Susanna and the Elders	Hayez	19	Susanna	painting	sitting	back	0.766	**0.706**
Venus	Ingres	19	Venus	painting	standing	front	0.778	**0.714**
The Birth of Venus	Keller	19	Venus	sculpture	sitting	front	0.818	**0.748**
Venus	Mayer	19	Venus	painting	lying	profile	0.797	**0.732**
The Birth of Venus	Pradier	19	Venus	sculpture	lying	front	0.723	**0.676**
Psyché enlevée par des Zéphyrs	Prud Hon	19	Psyche	painting	lying	profile	0.818	**0.752**
Vénus au bain ou l'Innocence	Prud Hon	19	Venus	painting	sitting	front	0.823	**0.740**
The Birth of Venus	Rutxhiel	19	Venus	sculpture	standing	front	0.806	**0.753**
Venus lifting drapery	Simart	19	Venus	sculpture	standing	front	0.742	**0.693**
Susanna and the Elders	Tassaert	19	Susanna	painting	standing	profile	0.814	**0.748**
Cupid and Psyche	Thorvaldsen	19	Psyche	sculpture	standing	front	0.834	**0.780**
Venus with apple	Thorvaldsen	19	Venus	sculpture	standing	front	0.794	**0.742**
The three Graces	Thorvaldsen	19	Grace	sculpture	standing	profile	0.807	**0.754**
The power of Venus	Westall	19	Venus	painting	lying	front	0.789	**0.725**

The Birth of Venus	Amaury-Duval	19.5	Venus	painting	standing	front	0.758	**0.696**
La Vague et la Perle	Baudry	19.5	Venus	painting	lying	back	0.771	**0.726**
Venus anadyomene	Bocklin	19.5	Venus	painting	standing	front	0.799	**0.734**
The Birth of Venus	Bouguereau	19.5	Venus	painting	standing	front	0.750	**0.688**
Love and Psyche	Bouguereau	19.5	Psyche	painting	lying	front	0.778	**0.714**
Cupid and Psyche	Brocky	19.5	Psyche	painting	lying	front	0.777	**0.714**
The Birth of Venus	Cabanel	19.5	Venus	painting	lying	front	0.757	**0.695**
Susanna and the Elders	Cabet	19.5	Susanna	painting	sitting	profile	0.783	**0.703**
Adam and Eve	Collier	19.5	Eve	painting	standing	profile	0.716	**0.657**
Venus	Corot	19.5	Venus	painting	sitting	profile	0.812	**0.730**
Vénus poursuit Psyché de sa jalousie	Courbet	19.5	Venus	painting	lying	front	0.770	**0.707**
Susanna and the Elders	Dupuis	19.5	Susanna	painting	sitting	profile	0.837	**0.753**
The Birth of Venus	Gerome	19.5	Venus	painting	standing	front	0.720	**0.660**
The chaste Susanna	Henner	19.5	Susanna	painting	standing	profile	0.798	**0.733**
Adam and Eve	Jones	19.5	Eve	painting	standing	front	0.817	**0.750**
The three Graces	Jones	19.5	Grace	painting	standing	back	0.754	**0.710**
Venus	Klimt	19.5	Venus	painting	standing	front	0.740	**0.679**
Cupid and Psyche	Legros	19.5	Psyche	painting	lying	front	0.770	**0.707**
The Bath of Psyche	Leighton	19.5	Psyche	painting	standing	profile	0.772	**0.709**
Venus	Leighton	19.5	Psyche	painting	standing	front	0.764	**0.702**
Adam and Eve	Machell	19.5	Eve	painting	standing	profile	0.782	**0.718**
Adam and Eve	Moreau	19.5	Eve	painting	standing	front	0.824	**0.757**
Susanna and the Elders	Moreau	19.5	Susanna	painting	standing	profile	0.836	**0.768**
Judgment of Paris	Parrot	19.5	Venus	painting	standing	front	0.787	**0.723**
Venus	Picou	19.5	Venus	painting	lying	front	0.748	**0.686**
Susanna and the Elders	Ranson	19.5	Susanna	painting	standing	front	0.809	**0.743**
The Toilet of Venus	Rodin	19.5	Venus	sculpture	standing	front	0.747	**0.698**
The Birth of Venus	Selous	19.5	Venus	painting	lying	front	0.776	**0.713**
Venus	Stott	19.5	Venus	painting	standing	front	0.769	**0.706**
Cupid and Psyche	Swynnerton	19.5	Psyche	painting	standing	front	0.756	**0.693**
The three Graces	Valtat	19.5	Grace	painting	standing	front	0.757	**0.695**
Smiling Venus	Dali	20	Venus	painting	lying	profile	0.824	**0.757**
Venus with Cupids	Dali	20	Venus	painting	sitting	back	0.835	**0.770**

Loved Psyche	Denis	20	Psyche	painting	standing	front	0.765	**0.703**
The three Graces	Girieud	20	Grace	painting	standing	front	0.781	**0.717**
The Birth of Venus	Redon	20	Venus	painting	standing	front	0.780	**0.717**
Judgment of Paris	Renoir	20	Venus	sculpture	standing	profile	0.793	**0.741**
The Awakening of Psyche	Seignac	20	Psyche	painting	standing	front	0.715	**0.655**
Judgment of Paris	Simonet	20	Venus	painting	standing	front	0.767	**0.704**
Adam and Eve	Valadon	20	Eve	painting	standing	front	0.751	**0.689**
Susanna and the Elders	Vallotton	20	Susanna	painting	standing	front	0.830	**0.763**
Venus	van Loo	20	Venus	painting	standing	front	0.750	**0.689**
Venus	Watson	20	Venus	painting	standing	front	0.735	**0.675**
The three Graces	Watts	20	Grace	painting	standing	front	0.842	**0.775**
Venus	Bravo	20.5	Venus	painting	lying	profile	0.739	**0.678**
Sleeping Venus	Delvaux	20.5	Venus	painting	lying	profile	0.736	**0.676**
The three Graces	Moser	20.5	Grace	painting	standing	profile	0.792	**0.727**
Susanna and the Elders	Zaech	20.5	Susanna	painting	standing	front	0.781	**0.718**
Cupid and Psyche	Andersson	21	Psyche	painting	standing	back	0.720	**0.678**
Judgment of Paris	Cassez	21	Venus	painting	sitting	front	0.766	**0.687**
Susanna and the Elders	Chambon	21	Susanna	painting	lying	profile	0.735	**0.675**
Psyche's Doubt	Watwood	21	Psyche	painting	sitting	front	0.751	**0.673**
